# Fabrication and Characterization of Short Silicon Nitride Fibers from Direct Nitridation of Ferrosilicon in N_2_ Atmosphere

**DOI:** 10.3390/ma11102003

**Published:** 2018-10-17

**Authors:** Jiasuo Guan, Yaohui Wang, Laifei Cheng, Yupeng Xie, Litong Zhang

**Affiliations:** 1 Science and Technology on Thermostructural Composite Materials Laboratory, Northwestern Polytechnical University, Xi’an 710072, China; guanjs@xaaq.com (J.G.); ahui88920@126.com (Y.W.); xieyupeng1981@163.com (Y.X.); zhanglt@nwpu.edu.cn (L.Z.); 2 Inner Mongolia Synthetic Chemical Engineering Institute, Hohhot 010010, China

**Keywords:** ferrosilicon, direct nitridation, silicon nitride fiber, vapor–liquid–solid mechanism

## Abstract

Short silicon nitride fibers were fabricated by direct nitridation of ferrosilicon in N_2_ atmosphere, and their structure and possible growth mechanism were characterized and investigated. The rod-like fibers which were α-Si_3_N_4_ with a low degree of crystallization and a high aspect ratio had a diameter of about 4 μm and a length close to a few millimeters. Belt-like fibers with a width about 5 μm and a thickness about 1 μm were also found in the nitrides. Scanning electron microscope (SEM), transmission electron microscope (TEM), high resolution transmission electron microscope (HRTEM), and selected area electron diffraction (SAED) investigations indicated that the fibers were single-crystalline α-Si_3_N_4_ with few amorphous distributed in the edge region, and the fibers grew by vapor–liquid–solid (VLS) mechanism.

## 1. Introduction

Silicon nitride fibers have been intensively studied due to its excellent mechanical properties, high thermal shock resistance and oxidation resistance, good electrical insulation, and high Young modulus and are useful as heat resistant, highly insulating materials or reinforcing agents for composites, particularly in the aerospace industry [1,2,3,4]. Another important application of Si_3_N_4_ nanomaterials worth mentioning is they also could be used to prepare nanofluids as presented in recent papers [5,6].

Generally, Si_3_N_4_ fibers can be commonly fabricated by pyrolysis of polymer precursors such as polysilazane (PSZ), polycarbosilazane (PCSZ) and polycarbosliane (PCS) [1]. Silicon nitride fibers were synthesized from the PCS by pyrolysis in ammonia gas atmosphere as early as in 1980s [7,8]. Si_3_N_4_ fibers, nanowires and nanobelts can also be synthesized via carbothermal reduction and nitridation of diatomaceous earth [9] or rice husk ash [10] or silica nanopowders and silica gel [11], laser assisted chemical vapor deposition (LCVD) process [12], Ni-catalyzed chemical vapor deposition (CVD) process [13], directly reaction between silicon and ammonia [14] or with catalyst assist [15].

In addition to the synthetic routes mentioned above, simple and economical methods for producing silicon nitride fibers from relatively inexpensive ferrosilicon (FeSi) alloy have been discovered and reported. T.E. Warner et al. [16] successfully synthesized Si_3_N_4_ fibers using ferrosilicon alloy and ammonia gas for first time in 2000, but the mechanism involved was not fully understood. Furthermore, nano-Si_3_N_4_ fibers were synthesized by Kaifu Huo et al. [17] in Nanjing University using ferrosilicon alloy powder and ammonia under 1300 °C.

However, synthesis of Si_3_N_4_ fibers using ferrosilicon alloy and nitrogen directly have rarely been reported. In this work, Si_3_N_4_ fiber was directly synthesized using ferrosilicon alloy powder and nitrogen at high temperature, and the microstructures and growth mechanism of silicon nitride fibers were investigated.

## 2. Materials and Methods

### 2.1. Raw Materials

The FeSi75Al2 (ISO 5445-80) ferrosilicon with a particle size less than 75 μm and a silicon content of 75% (Ningxia Puhua Metallurgical Products Co., Ltd., Qingtongxia, China) and nitrogen gas (99.999% purity, Xi’an Weiguang Gas Co., Ltd., Xi’an, China) were used as the main raw materials while self-made ferrosilicon nitride (Nitrogen content ≈ 30%) served as diluents. The element composition of FeSi75Al2 ferrosilicon were shown in Table 1, as our previous work [18] reported, the ferrosilicon alloy was a two-phase material consisting of silicon and high-temperature lebeauite FeSi_2_, and the role of ferrosilicon nitride diluents consist of α-Si_3_N_4_, β-Si_3_N_4_, Fe_3_Si, and some residual free silicon according to the results of X-ray diffraction (XRD) analysis is to decrease the reaction rate and prevent the melt droplets coalescence which results in low nitridation degree.

### 2.2. Preparation Process

The FeSi75Al2 alloy and self-made ferrosilicon nitride were first blended homogeneously according to a certain proportion, then mixed with polyvinyl alcohol (PVA) solution (5 wt %) which served as binder. The as-received mixtures then cold pressed into green bodies with a dimension of 70 × 15 × 4 mm^3^. Before nitridation, the samples were dried at a temperature of 150–200 °C to remove moisture and volatile impurities. The as-prepared green samples were laid separately in a graphite crucible, then placed into the sintering furnace (ECM Industrial Furnaces, 46, rue Jean Vaujany-TECHNISUD-38029 GRENOBLE CEDEX 2-FRANCE, Grenoble, France) for subsequent nitridation. The furnace was first hermetically vacuumized, then heat up with a heating rate about 2~3 °C per minute, high purity nitrogen gas was filled with a pressure range from 0.03 MPa to 0.05 MPa when the temperature in the furnace reaches 900 °C, then holding a certain time at 1140, 1350 and 1450 °C in turn. The temperature was measured by a thermocouple inserted in the furnace. After nitridation, the samples were cooled down naturally to room temperature begin from 200 °C in nitrogen and then extracted from the furnace for further investigations.

### 2.3. Characterization

Morphology and EDS analysis of the nitride products was investigated by scanning electron microscope (SEM, S-4700, Hitachi, Tokyo, Japan) and energy dispersive X-ray spectroscopy (EDS, Genesis XM2, EDAX, Mahwah, NJ, USA). The microstructure of the nitrides was also observedby transmission electron microscope (TEM, G-20, FEI-Tecnai, Hillsboro, OR, USA).The phase compositions of reaction products were determined by an X-ray diffractometer (Cu K_α_, D8 ADVANCE, Bruker, Billerica, MA, USA).

## 3. Results

### 3.1. Morphology and Microstructure

**Figure 1:**
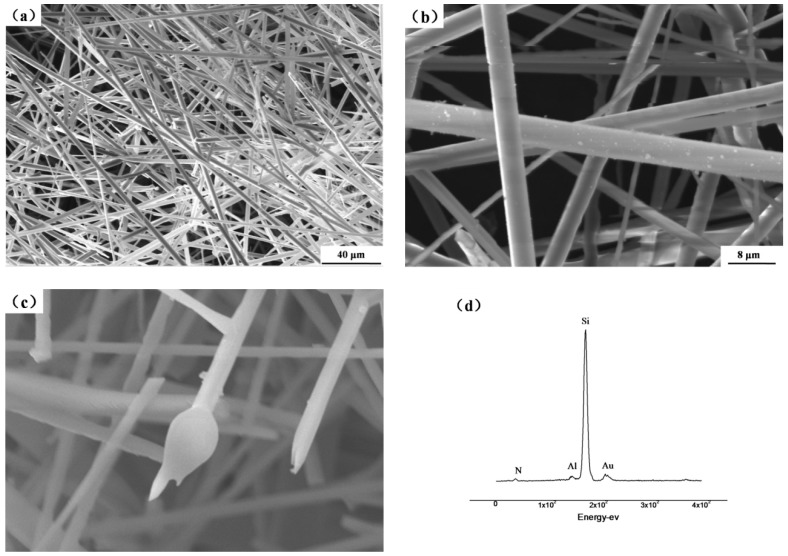
(**a**,**b**) Morphologies of short fiber nitride products, (**c**) morphology and (**d**) EDS analysis of the droplets.

After the nitridation process of ferrosilicon alloy in nitrogen atmosphere, amount of white fibers was found on the surface of the products and on the walls of the graphite crucible. The SEM and EDS results showed in Figure 1 indicate that the silicon nitride fiber does not align in the same orientation, but along different directions. The diameter of each fiber is about 4 μm while the length of the whole fiber which cannot be determined from the microstructure image but can be deduced from the macrostructure image that is about few millimeters. Therefore, this silicon nitride fiber has a high aspect ratio.

**Figure 2:**
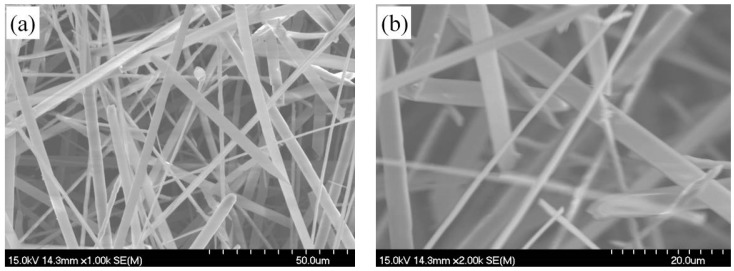
Micromorphologies of α-Si_3_N_4_ belts (**a**) 50.0 μm; (**b**) 20.0 μm.

Besides those rod-like silicon nitride fibers mentioned above, ribbon-like fiber can also be seen in the SEM image, as shown in Figure 2. The narrow and thin ribbon-like fibers maintain a consistent width and thickness along the length direction which are about 5 μm and 1 μm, respectively. Therefore, the morphology of the silicon nitride fibers is also diverse.

**Figure 3:**
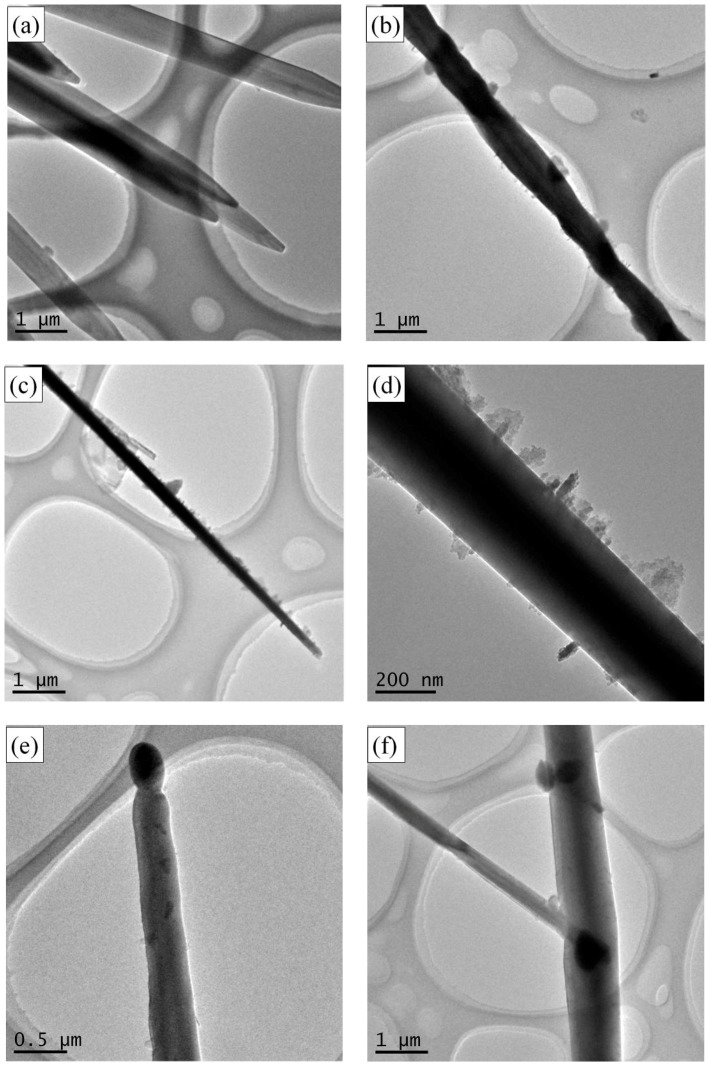
TEM images of fiber-like nitride products. (**a**) overlapped, (**b**) twist and (**c**,**d**) rough features, (**e**,**f**) droplets of fibers.

The microstructure of Si_3_N_4_ fiber was also studied, as presented in Figure 3. Figure 3a shows the ribbon-like silicon nitride fibers were overlapped, but the copper sample holder can still be seen clearly, indicating that the thickness of the fiberwas low and the fibercan be penetrated by electrons. The fiber showed in Figure 3b has twist feature, illustrating that feature of the fibers were diverse. As can be seen in Figure 3c,d, the width of the fiber became smaller and the surface became rough along the direction to the top.

It also can be seen that impurity appeared on both sides of the fiber. As also could be seen in Figure 3e,f, droplets appeared in the fiber end, which was a typical feature of vapor–liquid–solid (VLS) growth mechanism.

**Figure 4:**
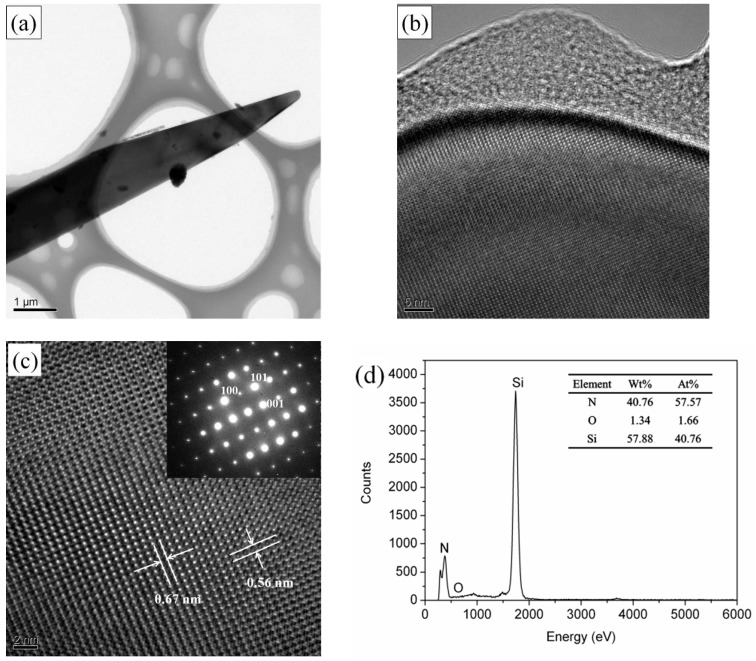
TEM images of the α-Si_3_N_4_ fibers. (**a**) A typical low-magnification TEM image. (**b**) HRTEM images of α-Si_3_N_4_ fibers. (**c**) HRTEM images and corresponding SAED patterns (inset pictures). (**d**) A typical EDS pattern obtained from a α-Si_3_N_4_ fiber.

The TEM image, HRTEM image, SAED image, and EDS spectrum of Si_3_N_4_ fibers were included in Figure 4. Figure 4b shows that the end of the fiber has amorphous characteristics, and its interior is crystalline. According the HRTEM and corresponding SAED diagrams presented in Figure 4c, the crystallized fiber is a single crystal without any defectssuch as dislocation or stacking fault. The average spacing of the lattice fringe is 0.67 nm and 0.56 nm, respectively, which is consistent with the crystal surface spacing of the crystal plane (100) and (001) of the hexagonal α-Si_3_N_4_.The atomic ratio of Si and N from EDS spectrum observed in Figure 4d is similar to atomic ratio of Si_3_N_4_, which further indicates the fiber is Si_3_N_4_. The amorphous region shown in Figure 4b could be silicon oxide, due to the oxygen detected from the EDS spectrum which may come from nitrogen or residual air in the furnace. The oxygen impurity can be removed by hydrogen gas in future work to improve the purity of silicon nitride fiber.

### 3.2. XRD Characterization

**Figure 5:**
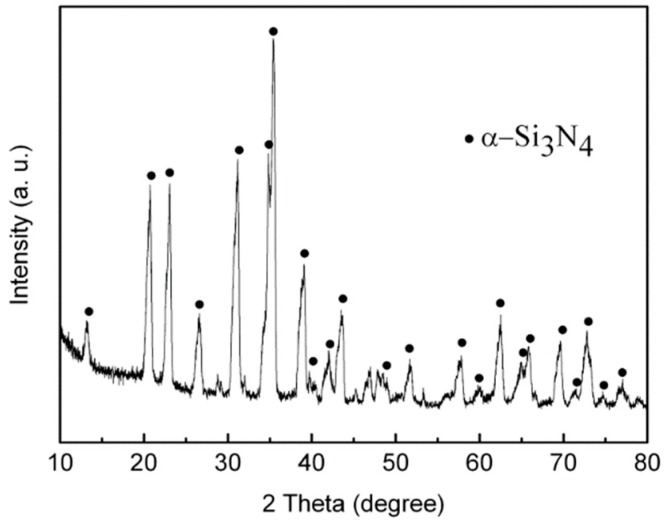
X-ray diffraction pattern of the fiber-like nitride products.

XRD (Figure 5) analyses indicate that the fibers are α-Si_3_N_4_ with a low degree of crystallization. As mentioned in our previous work [19], appearance of low-temperature α-modification silicon nitride with a low degree of crystallization was attributed to the lower temperature of the wall of the crucible and the surface of the samples compared to the heart of them. Detailed studies of the fiber composition, purity, and process condition optimization are being planned and carried out.

### 3.3. Growth Mechanism

From SEM images exhibited in Figure 1b,c and TEM images showed in Figure 3e,f, it can be seen that spherical droplets appeared at the tips of the fibers. Themicroscopic feature revealed the characteristics of the VLS mechanism [14,19,20,21]. The liquid phase of ferrosilicon alloy necessary for VLS growth appearedwhen FeSi_2_-Si eutectic melts at 1206 °C which is much lower than melting point of pure silicon [22]. Therefore, the possible growth mechanism of silicon nitride fiber is VLS mechanism.

According to the typical characteristics of VLS growth mechanism, the growth process of silicon nitride fiber generated by ferrosilicon alloy can be divided into the following three stages:

In the initial stage, the liquid eutectic Fe-Si phase formed as a droplet at 1206 °C [22] which is much lower than the nitradation temperature. Such a liquid droplet is a preferred site for deposition of Si vapor and N_2_ [19], then the Si vapor and N_2_ in the high temperature diffused into Fe-Si droplets, further forming eutectic Fe-Si-N liquid droplets [23].

In the following stage, as the absorption–dissolution of silicon and nitrogen particles proceed, a large amount of Si-N phase dissolved into the droplet. When the Fe-Si-N droplet is supersatuated with Si-N, α-Si_3_N_4_ nucleus start to form.

Finally, a solid–liquid interphase is formed between α-Si_3_N_4_ nucleus and the Fe-Si-N droplet. As the reaction between Si and N continues, the Si_3_N_4_ concentration increase until exceeds the saturationlevel of Fe-Si-N droplet, then the supersaturated silicon nitride was separated out at the solid–liquid interphase. Thus a silicon nitride concentration gradient formed, so the silicon nitride constantly diffused from the high concentration side to the solid–liquid interphase which is the low silicon nitride concentration side. As well as, the Gibbs free energy for the silicon nitride to separate out at the solid–liquid interphase was lower than forming a new solid–liquid phase. Therefore, the silicon nitride fibers continuously grow in one direction until silicon particles fully react with nitrogen.

**Figure 6:**
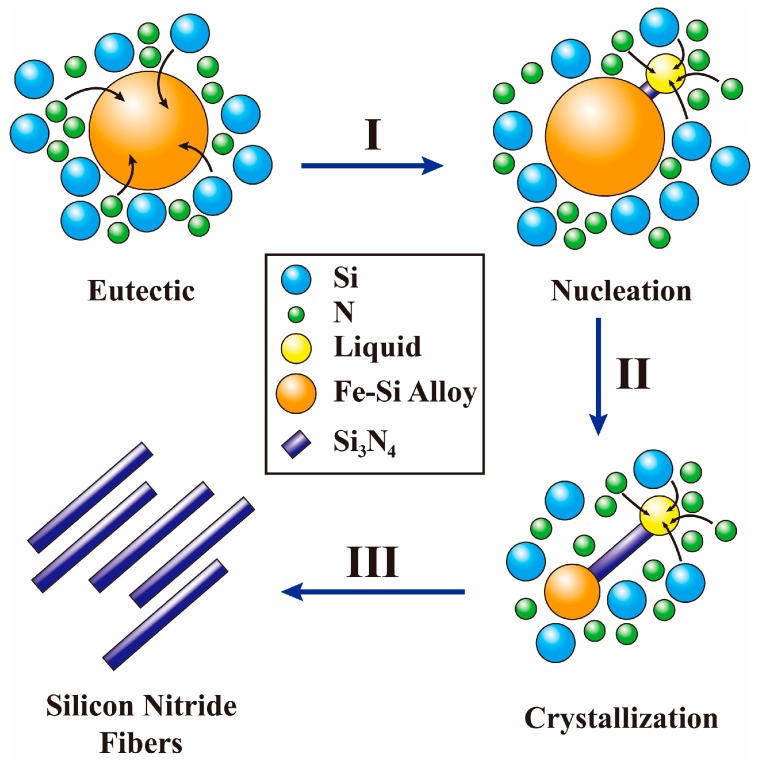
Schematic diagram for the growth of α-Si_3_N_4_ fibers.

The growth process can be simply illustratedin Figure 6.

## 4. Conclusions

A novel synthestic method for Si_3_N_4_ fiber was found in this study. Si_3_N_4_ fiber can be synthetized by direct nitriding ferrosilicon in nitrogen atmosphere between 1150 °C and 1450 °C. The diameter of a single rod-like fiber is about 3 μm, and the length of a fiber was about a few millimeters, hence the fibers have a high value of aspect ratio. In addition of the rod-like fibers, ribbon-like fibers with similar length were also observed. These ribbon-like fibers have a width about 5 μm and a thickness about 1 μm. The TEM images showed that the fibers are single crystal with small amount of amorphous structure at the edge. Droplet structure was obtained at the tip of fibers. The fibers grew by VLS mechanism. The iron in ferrosilicon promotes the forming of liquid droplet at a relatively low temperature which is necessary for VLS growth.

## Figures and Tables

**Table 1:** Elemental composition of FeSi75Al2 alloy.

Element	Si	Fe	Al	C	P	S
Mass fraction/wt %	75.76	22.55	1.58	0.076	0.029	0.0034

## References

[1] Li Y., Gao J. (2013). Preparation of silicon nitride ceramic fibers from polycarbosilane fibers by γ-ray irradiation curing. Mater. Lett..

[2] Hu X., Shao C., Wang J., Wang H., Cheng J. (2017). Effects of residual radicals on compositional and structural stability of silicon nitride fibers. J. Eur. Ceram. Soc..

[3] Hu X., Shao C., Wang J., Wang H. (2017). Characterization and high-temperature degradation mechanism of continuous silicon nitride fibers. J. Mater. Sci..

[4] Arai M., Osamu F., Hayato N., Takeshi I. (1989). High-Purity Silicon Nitride Fibers. U.S. Patent.

[5] Żyła G., Vallejo J.P., Lugo L. (2018). Isobaric heat capacity and density of ethylene glycol based nanofluids containing various nitride nanoparticle types: An experimental study. J. Mol. Liq..

[6] Żyła G., Fal J., Bikić S., Wanic M. (2018). Ethylene glycol based silicon nitride nanofluids: An experimental studyon their thermophysical, electrical and optical properties. Phys. E Low-Dimens. Syst. Nanostruct..

[7] Taki T., Okamura K., Sato M., Seguchi T., Kawanishi S. (1988). A study on the electron irradiation curing mechanism of polycarbosilane fibers by solid-state ^29^Si high-resolution nuclear magnetic resonance spectroscopy. J. Mater. Sci. Lett..

[8] Taki T., Inui M., Okamura K., Sato M., Seguchi T. (1991). A study of nitridation process of polycarbosilane fibers by solid-state high-resolution NMR. Appl. Magn. Reson..

[9] Mizuhara Y., Noguchi M., Ishihara T., Satoh A., Hiramatsu K., Takita Y. (1991). Preparation of Fiber like Silicon Nitride from Diatomaceous Earth. J. Am. Ceram. Soc..

[10] Pavarajarn V., Precharyutasin R., Praserthdam P. (2010). Synthesis of silicon nitride fibers by the carbothermal reduction and nitridation of rice husk ash. J. Am. Ceram. Soc..

[11] Deshmukh S., Jen K.P., Santhanam S. (2012). Comparison of silicon nitride nanofibers synthesized using silica nanopowders and silica gel. Mater. Sci. Appl..

[12] Wallenberger F.T., Nordine P.C. (1994). Amorphous silicon nitride fibers grown from the vapor phase. J. Mater. Res..

[13] Huang J., Zhang S., Huang Z., Liu Y.G., Fang M. (2013). Growth of α-Si_3_N_4_ nanobelts via Ni-catalyzed thermal chemical vapour deposition and their violet-blue luminescent properties. CrystEngComm.

[14] Gopalakrishnan P.S., Lakshminarasimham P.S. (1993). Preparation of fiber-like silicon nitride from silicon powder. J. Mater. Sci. Lett..

[15] Kim H.Y., Park J., Yang H. (2003). Synthesis of silicon nitride nanowires directly from the silicon substrates. Chem. Phys. Lett..

[16] Warner T.E., Fray D.J. (2000). Synthesis of silicon nitride fibers from ferrosilicon. J. Mater. Sci. Lett..

[17] Huo K., Ma Y., Hu Y., Fu J., Lu B., Lu Y., Hu Z., Chen Y. (2005). Synthesis of single-crystalline α-Si_3_N_4_ nanobelts by extended vapour-liquid-solid growth. Nanotechnology.

[18] Wang Y., Cheng L., Guan J., Zhang L. (2014). Effect of dilution and additive on direct nitridation of ferrosilicon. J. Eur. Ceram. Soc..

[19] Wagner R.S., Ellis W.C. (1964). Vapour-Liquid-Solid mechanism of single crystal growth. Appl. Phys. Lett..

[20] Morales A.M., Lieber C.M. (1998). A Laser Ablation Method for the Synthesis of Crystalline Semiconductor Nanowires. Science.

[21] Kukovitsky E.F., L’vov S.G., Sainov N.A. (2000). VLS-growth of carbon nanotubes from the vapor. Chem. Phys. Lett..

[22] Chukhlomina L.N., Maksimov Y.M. (2008). Effect of the silicon content in the initial alloy on synthesis of silicon nitride in burning ferrosilicon in nitrogen. Glass Ceram..

[23] Huang J., Huang Z., Yi S., Liu Y., Fang M., Zhang S. (2013). Fe-catalyzed growth of one-dimensional α-Si_3_N_4_nanostriuctures and their cathodoluminescence properties. Sci. Rep..

